# Sleep Disorders and Their Relationship With Temporomandibular Joint Disorders Derived From a Psychoemotional Disorder

**DOI:** 10.1155/crid/1711912

**Published:** 2026-05-11

**Authors:** Franklin Andrés Cardoso Zumba, Gilma Katherine Chamba López, Juan Alejandro Jiménez Encalada, Wilson Daniel Torres Bravo

**Affiliations:** ^1^ Faculty of Dentistry, Universidad Católica de Cuenca, Cuenca, Ecuador; ^2^ Faculty of Dentistry, Universidad de Cuenca, Cuenca, Ecuador, ucuenca.edu.ec

**Keywords:** orofacial pain, osteoarthritis, osteoarthritis, sleep disorders, TMJ

## Abstract

Sleep disorders are considered a significant risk factor for the onset and development of temporomandibular disorders (TMDs); additionally, sleep disorders contribute to generating a proinflammatory state and central sensitization, which can cause temporomandibular joint (TMJ) pain. A 50‐year‐old female patient reported pain near the left ear to the dental clinic. Her medical history includes a sleep disorder, stress, and anxiety. After analyzing her medical records, clinical findings, and imaging studies, an inflammatory‐degenerative process was diagnosed in the left TMJ. The treatment administered was arthrocentesis, which relieved the pain for a period of 13 months. This clinical case report is aimed at highlighting the association between sleep disorders and TMDs, to generate multidisciplinary and transdisciplinary clinical protocols that lead to accurate diagnosis and efficient treatment plans.

## 1. Introduction

Temporomandibular disorders (TMDs) is a term that encompasses musculoskeletal conditions involving pain and/or dysfunction in the masticatory muscles, temporomandibular joints (TMJs), and associated structures [1, 2]. In 2018, the American Academy of Orofacial Pain updated the classification of TMDs, providing the best available reference for clinical and research purposes [3]. (Table 1).

**Table 1:** Taxonomy of temporomandibular disorders, 2018.

TMJs disorders	Masticatory muscles disorders	Headaches	Associated structures
1. Joint pain: a. Arthralgia b. Arthritis 2. Joint disorders: a. Disc displacement with reduction b. Disc displacement with reduction and intermittent locking c. Disc displacement without reduction and limited opening d. Disc displacement without reduction and without limited opening 3. Joint diseases: a. Degenerative joint disease *i. Osteoarthritis* *ii. Osteoarthrosis* b. Condylosis c. Osteochondritis dissecans d. Osteonecrosis e. Systemic arthritis f. Neoplasia g. Synovial chondromatosis 4. Fractures a. Closed fracture of the condylar process b. Closed fracture of the sub‐condylar process c. Open fracture of the condylar process d. Open fracture of the sub‐condylar process 5. Congenital/developmental joint disorders a. Condylar aplasia b. Condylar hypoplasia c. Condylar hyperplasia	1. Muscle pain: a. Myalgia i. *Local myalgia* ii. *Myofascial pain* iii. *Myofascial pain with referral* b. Tendinitis c. Myositis i. *Noninfectious* ii. *Infectious* d. Spasms 2. Contracture: a. Muscle b. Tendon 3. Hypertrophy. 4. Neoplasms: a. Jaw i. *Malignant* ii. *Benign* b. Soft tissues of the head, face, and neck i. *Malignant* ii. *Benign* 5. Movement disorders: a. Orofacial dyskinesia i. *Abnormal involuntary movements* ii. *Unspecified ataxia and lack of muscle coordination* iii. *Subacute*, *drug-induced*; *tardive oral dyskinesia* b. Oromandibular dystonia i. *Acute drug-induced* ii. *Deforming dystonia*, *familial*, *idiopathic*, *and torsion dystonia* 6. Pain in the masticatory muscles attributed to systemic/central pain disorders: a. Fibromyalgia b. Central medication‐induced myalgia	1. Headaches attributed to TMD.	1. Coronoid hyperplasia

Painful TMDs arise from complex and dynamic interactions among physiological, psychological, and social factors that can maintain and amplify pain and disability [4], with psychological symptoms contributing most significantly to the initial onset of TMDs [2]. These painful TMDs are often associated with comorbidities, including migraines, tension headaches, depression, sleep disorders, degenerative arthritis, chronic fatigue, dizziness, tinnitus, gastrointestinal issues, and allergies. Therefore, the disorder does not appear to occur in isolation [5, 6].

Among the physical and psychological factors, disrupted and nonrestorative sleep has been identified as a significant risk factor for the development of new‐onset TMDs or flare‐ups of symptoms in individuals with chronic TMDs [5, 7]. This sleep loss contributes to a proinflammatory state or central sensitization, which increases pain processing [5]. The direct relationship between TMJ pain intensity and poor sleep quality occurs because sleep fragmentation disrupts circadian cycles, increasing circulating levels of inflammatory cytokines that trigger catabolic responses and the progression of TMDs [8].

Lermman et al. reported that women with TMDs who suffer from insomnia and short sleep duration (ISSD) develop a severe clinical pain profile, functional limitation of the jaw, greater pain sensitivity, and central sensitization [8].

Given the above, the purpose of this article is to analyze a clinical case related to sleep disorders and TMDs, following CARE publication guidelines.

## 2. Case Report

A 50‐year‐old female patient presented to a dental consultation, reporting pain near the left ear. She stated that she could not recline more than 45° due to episodes of vertigo and anxiety accompanied by pain near the left ear, symptoms that had persisted for over 4 years.

In her medical history, the patient mentioned that she had consulted with specialists who conducted CT scans and MRI of the head and ear to reach a diagnosis in search of the origin of dizziness, seizures, joint pain, sleep disorders, and nyctophobia (fear of the dark). All tests showed no medical abnormalities, and the treatments provided by professionals did not result in any improvement, although she received treatment with anxiolytics and other psychotropic derivatives that masked the symptoms. The patient also mentioned that she consulted with a psychologist who diagnosed stress and anxiety.

The patient visited the dental clinic as a last resort; her primary complaint was pain in the left side of the joint, which had persisted for more than 4 years. A clinical examination of the TMJ was performed through inspection, palpation, and auscultation, revealing pain in the lateral pterygoid muscles, predominantly on the left side. There was also a jaw deviation to the left upon opening, accompanied by joint noises consistent with unilateral left‐sided crepitus.

**Figure 1:**
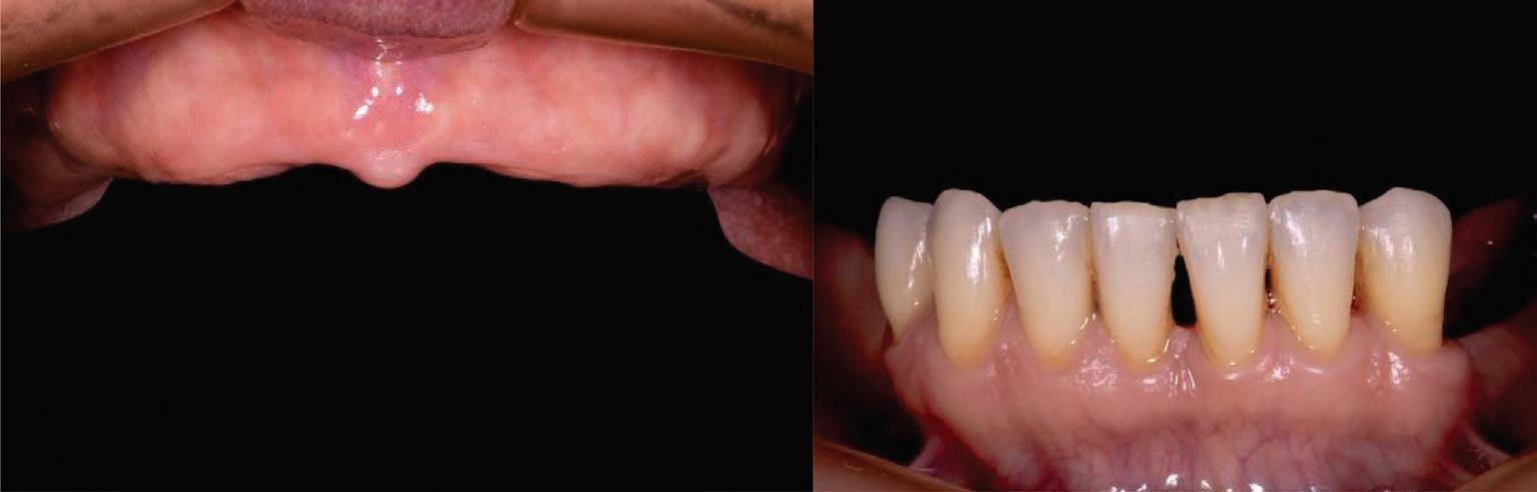
Upper jaw: completely edentulous for more than 5 years. Lower jaw: Kennedy Class 1 partial edentulism.

In the intraoral examination, the patient showed a reduced vertical occlusal dimension (VOD) due to the loss of her dental organs, with a completely edentulous upper jaw and a partially edentulous lower jaw classified as Kennedy Class I (Figure 1) for more than 7 years, according to the anamnesis. She had a 5‐year‐old upper complete denture showing signs of wear.

**Figure 2:**
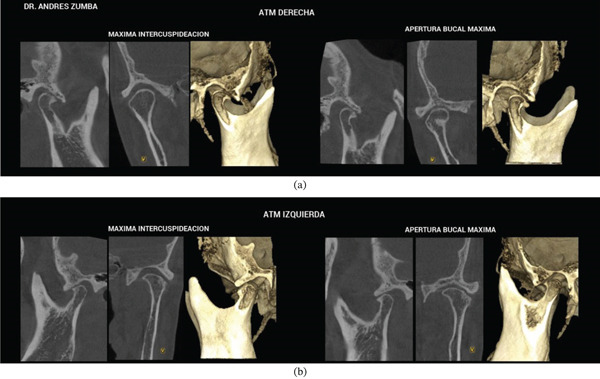
(a) Right TMJ. Maximum intercuspation and maximum opening; presence of osteophyte products from subchondral cysts, slightly compromised condylar body. (b) Left TMJ. Maximum intercuspation and maximum opening; degradation of the mandibular condyle′s cortical bone; and presence of an osteophyte resulting from superficial bone erosion due to a subchondral cyst.

Given the clinical findings, the presence of a temporomandibular disorder was evident. Tomographic studies of the joint were requested with the mouth open and closed (Figure 2), revealing the following:

In the sagittal view, condylar deformation was observed, with the left condyle showing marked deformation, presenting a 2‐mm osteophyte and a subchondral cyst. According to the classification by Wilkes, the patient was in Stage IV, associated with joint degeneration [9].

**Figure 3:**
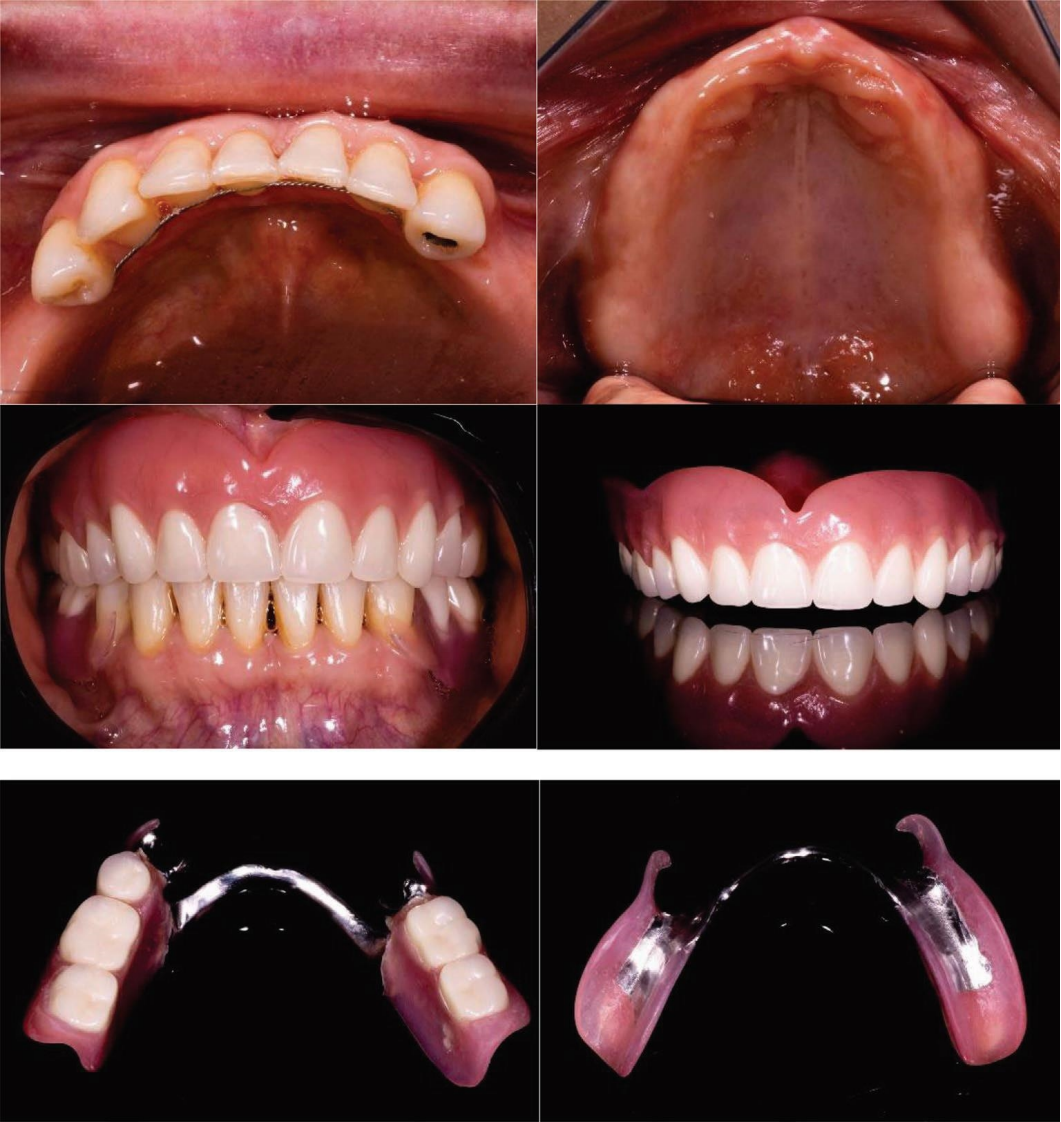
Alignment of the lower anterior teeth and fabrication of working removable prostheses to evaluate symptoms in the short term.

As a treatment plan, an attempt was made to improve the patient′s occlusal stability by aligning the lower anterior teeth to rehabilitate with a provisional lower partial denture and a provisional upper complete denture, with a 2‐mm increase in the VOD. This increase, in addition to its aesthetic purpose, is aimed at decompressing the joint capsule and establishing a new occlusal scheme with greater masticatory efficiency (Figure 3).

For differential diagnosis, a wet puncture with anesthesia was performed at the left auricular nerve level with 2% lidocaine with epinephrine 1:80000 (Xylestesin A). The patient reported a sensation of relief, and her mouth opening increased from 3 to 4 cm. She could recline fully in the dental chair, and days later, the patient reported reduced pain and vertigo.

After confirming that it was an inflammatory‐degenerative process in the left TMJ (osteoarthritis), an articular lavage (arthrocentesis) was performed [10] with physiological saline and an injectable solution of betamethasone (Betamethasone 6 mg/2 mL–betamethasone acetate 6 mg/2 mL). Analgesic and anti‐inflammatory medication was prescribed, dietary recommendations were given (soft diet), and the patient was observed while her new prostheses were made to stabilize the occlusal plane.

**Figure 4:**
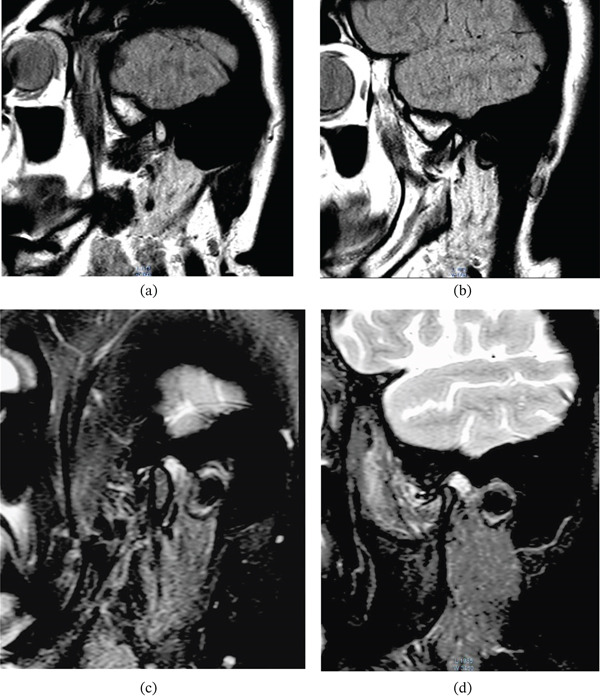
(a) T1 closed mouth (right). (b) T1 closed mouth (left). In both cases, anterior disc displacement is observed. (c) T2 open mouth (right). (d) T2 open mouth (left). In both cases, nonreducible disc displacement is observed, with the presence of inflammatory fluid.

The patient reported relief and a reduction in vertigo for about a year. However, she stated that the symptom of pain in the left auricular area had returned, although with less intensity, along with episodes of epilepsy and nyctophobia, after 13 months following the articular lavage and the installation of her upper complete denture and lower partial denture. A follow‐up MRI was requested, and a new articular lavage was planned. The imaging study indicated a nonreducible disc displacement in both joints, with inflammatory fluid in both joints, consistent with osteoarthritis in both joints (Figure 4). A new articular lavage of the TMJs was recommended to reduce the inflammatory process.

## 3. Discussion

TMDs are conditions that must be evaluated and treated based on scientific evidence. Treating TMDs empirically can worsen the patient′s current condition. With proper training in diagnosing and treating TMDs, an appropriate treatment protocol can be developed to improve patients′ quality of life.

TMDs affect 5%–12% of the general population, making it the second most common musculoskeletal condition after chronic low back pain, resulting in pain and disability. This disorder affects patients′ daily activities physically and psychosocially and reduces their quality of life [11].

Diagnostic methods for TMDs include clinical (auscultation, palpation, and anesthetic blocks) and imaging methods (x‐rays, CT scans, and MRIs) [12]. According to Schiffman et al., it is essential to focus on locating the etiology of TMDs based on clinical findings such as myalgias, myofascial pain, arthralgia, disc displacements, limited mouth opening, subluxations, and psychosocial findings such as patient distress and disability associated with pain to estimate the patient′s prognosis [11]. In parallel, Derwich et al., in their literature review, mentioned that degenerative changes in the TMJs are evident in imaging studies and are characterized by the presence of osteophytes (bone formations on the condylar surface); pseudocysts (osteophytes, well‐defined changes localized in the subcortical area); erosion (the area of reduced density within the cortex and subcortical bone); sclerosis (increased density of the cortical plate or bone tissue beneath the cortical plate); and flattening of the convex condylar head [13]. Wilkes presented a five‐stage classification that compares clinical symptoms with radiological findings of TMDs, being an effective diagnostic resource for visualizing degenerative changes in the course of TMJ osteoarthritis in tomographic images [9]. Currently, MRI is considered the gold standard in diagnosing the morphology and position of the TMJ disc [14], being an effective technique for visualizing degenerative changes in the course of TMJ osteoarthritis [15]. However, when degenerative changes are late, disc perforations may occur, which can be challenging to diagnose in MRI images [16]. In our clinical case report, the patient presented clinical findings consistent with the development of a temporomandibular disorder due to an overload in the TMJs caused by the loss of dental organs, with a lack of occlusal stability and mandibular imbalance.

For the treatment of inflammatory processes within the TMJ, particularly in patients whose standard noninvasive treatment protocols have not alleviated the clinical condition, minimally invasive surgical procedures such as arthrocentesis can be performed. Arthrocentesis involves flushing the upper compartment of the TMJ with a saline solution, with the main objective of precisely eliminating inflammatory mediators, releasing the disc, breaking adhesions, eliminating pain, and improving joint mobility. It is a method with minimal complications, simple, repeatable, and outpatient [17]. Arthrocentesis can also be combined with hyaluronic acid, corticosteroids, or platelet‐rich plasma injection [13]. Gencer et al. investigated the relief of discomfort in TMJ disorders from three intra‐articular injections: hyaluronic acid, tenoxicam, and betamethasone, observing significantly better pain scores in the examined groups compared with the control group (saline solution), with the hyaluronic acid group performing better than other anti‐inflammatory agents [18].

Publications such as that by Jiménez‐Silva et al., who presented a systematic literature review on the discrepancy of the centric relation‐intercuspation position and its relation to TMDs [19], indicate that there is no evidence supporting that centric relation discrepancies are related to TMDs. Therefore, in our clinical case, we did not intend to lead or induce the patient into a centric relation but rather attempted to establish a habitual position for the patient that is comfortable and does not cause pain.

In a systematic review published by Medeiros Veiga et al. on sleep quality in patients with TMJ disease or disorder, they found a strong association between sleep disorders and TMDs, and they focused on performing polysomnographies to diagnose the relationship between lack of sleep and pain caused by TMD [20]. Similarly, Sánchez Romero et al., in their systematic review of the association between sleep disorders and sleep quality in patients with TMJ osteoarthritis, concluded that due to the low quality of evidence, TMD cannot be directly related to a sleep disorder [7]. Therefore, more studies and long‐term follow‐ups are suggested to investigate if statistically significant relationships exist.

Taking into account the signs and symptoms with which the patient presented, the medical diagnostic processes the patient underwent, and the dental procedures performed, an improvement in the patient′s quality of life was achieved. The inflammatory process has indeed returned after 13 months, but given that the patient has sleep disturbances and emotional state alterations, this inflammatory process is expected. We hope that with the medical treatments for the sleep disturbances and emotional state, over time, the patient′s quality of life will improve permanently.

## 4. Conclusions

Painful TMDs are triggered by pathophysiological, traumatic, psychological, and social factors, which can lead patients to a state of chronic pain, resulting in a state of disability that will affect their quality of life. The aim of reporting this clinical case is to highlight the possible relationship between sleep disorders and psychological alterations with the presence and perpetuation of TMDs. Therefore, in such clinical cases, it is important to carry out multidisciplinary and transdisciplinary treatment with the sole purpose of achieving an integral diagnosis and improving the quality of life of our patients, all based on scientifically validated clinical protocols.

## Funding

No funding was received for this manuscript.

## Conflicts of Interest

The authors declare no conflicts of interest.

## Data Availability

Data sharing is not applicable to this article as no datasets were generated or analyzed during the current study.
